# Impact of lactate dehydrogenase on prognosis of patients undergoing cardiac surgery

**DOI:** 10.1186/s12872-022-02848-7

**Published:** 2022-09-10

**Authors:** Yu Zeng, Yuhe Zhao, Shuren Dai, Yanyan Liu, Ruoyu Zhang, Hong Yan, Min Zhao, Yong Wang

**Affiliations:** Department of Cardiology, Chongqing the Seventh People’s Hospital, Chongqing, China

**Keywords:** Cardiac surgery, Lactate dehydrogenase, Mortality, Complications, Medical Information Mart for Intensive Care

## Abstract

**Background:**

Lactate dehydrogenase (LDH) has been reported in multiple heart diseases. Herein, we explored the prognostic effects of preoperative LDH on adverse outcomes in cardiac surgery patients.

**Methods:**

Retrospective data analysis was conducted from two large medical databases: Medical Information Mart for Intensive Care (MIMIC) III and MIMIC IV databases. The primary outcome was in-hospital mortality, whereas the secondary outcomes were 1-year mortality, continuous renal replacement therapy, prolonged ventilation, and prolonged length of intensive care unit and hospital stay.

**Results:**

Patients with a primary endpoint had significantly higher levels of LDH (*p* < 0.001). Multivariate regression analysis presented that elevated LDH was independently correlated with increased risk of primary and secondary endpoints (all *p* < 0.001). Subgroup analyses showed that high LDH was consistently associated with primary endpoint. Moreover, LDH exhibited the highest area under the curve (0.768) for the prediction of primary endpoint compared to the other indicators, including neutrophil–lymphocyte ratio (NLR), lymphocyte-monocyte ratio (LMR), platelet–lymphocyte ratio (PLR), lactate, and simplified acute physiology score (SAPS) II. The above results were further confirmed in the MIMIC IV dataset.

**Conclusions:**

Elevated preoperative LDH may be a robust predictor of poor prognosis in cardiac surgery patients, and its predictive ability is superior to NLR, LMR, PLR, lactate, and SAPS II.

**Supplementary Information:**

The online version contains supplementary material available at 10.1186/s12872-022-02848-7.

## Introduction

In the United States, approximately 300,000 people undergo cardiac surgery each year, of which over 80% are coronary artery bypass graft (CABG) and valve surgeries [1]. Although mortality after cardiac surgery has decreased significantly over previous deCHDes due to the progress of cardiopulmonary bypass (CPB) and surface hypothermia [2–4], postoperative complications are still high [5]. Acute kidney injury is one of the most frequent complications of cardiac surgery, with an incidence rate ranging from 5 to 50% depending on variable definitions [6, 7]. In addition, low cardiac output syndrome after cardiac surgery has a high prevalence, with varying occurrences ranging from 10 to 20% [8]. Evidence suggests that these organ damages probably linked to systemic inflammatory response and ischemia–reperfusion injury caused by CPB [9]. Previous studies have shown that inflammatory markers, such as lymphocyte-to-monocyte ratio (LMR) and neutrophil–lymphocyte ratio (NLR), are connected with unfavorable outcomes in patients undergoing cardiac surgery [10, 11].

Long-term ischemia leads to insufficient oxygen supply and hypoxia. Lactate dehydrogenase (LDH) is a significant intracellular enzyme in energy production, which catalyzes pyruvate to lactate under anaerobic conditions [12]. Initially, the increase of LDH can reflect cardiac damage and is used to diagnose acute myocardial infarction [13]. Subsequently, LDH has been found to be elevated in patients with valve heart disease, heart failure, and coronary heart disease [14, 15]. However, no study has investigated the relationship between LDH and poor prognosis of patients undergoing cardiac surgery. Thus, we attempted to explored the prognostic impact of LDH at admission on poor prognosis in patients after cardiac surgery.

## Materials and methods

### Study design

This was a retrospective cohort study based on two large publicly available critical care databases the Medical Information Mart for Intensive Care III version 1.4 (MIMIC III v 1.4) and MIMIC IV v 0.4 [16, 17]. MIMIC-III covers over 40,000 intensive care unit (ICU) admissions at the Beth Israel Deaconess Medical Center in Boston between 2001 and 2012. MIMIC-IV is an update to MIMIC-III. We passed the Protecting Human Research Participants exam and obtain the seniority to access these databases. Data were extracted by authors YZ, YYL and YHZ.

### Population selection criteria

Adult patients who underwent cardiac surgery and were admitted to the ICU for the first time were included in the study. The following exclusion criteria were applied: (1) missing LDH data at admission, and (2) missing data > 5%.

### Data extraction

The extracted data contained age, gender, marital status, ethnicity, body mass index (BMI), heart rate, systolic blood pressure (SBP), diastolic blood pressure (DBP), simplified acute physiology score (SAPS) II, sequential organ failure assessment (SOFA) score, comorbidities, laboratory parameters and outcomes. Comorbidities comprised hypertension, diabetes, coronary heart disease (CHD), valve disease, heart failure (HF), chronic obstructive pulmonary disease (COPD), and chronic kidney disease (CKD). Laboratory parameters included LDH, white blood cell (WBC) count, platelet (PLT) count, blood urea nitrogen (BUN), serum creatine (SCr), sodium, potassium, glucose, neutrophil count, lymphocyte count, monocyte count, and lactate. We took the preoperative laboratory indicators from the first results after admission. The NLR, LMR, and platelet–lymphocyte ratio (PLR) were calculated as follows: NLR = neutrophil/lymphocyte counts, LMR = lymphocyte/ monocyte counts, and PLR = platelet/lymphocyte counts. Cardiac surgical procedures included CABG and/or heart valve surgery. The primary endpoint was in-hospital mortality, whereas the secondary endpoints were 1-year mortality, continuous renal replacement therapy (CRRT), prolonged ventilation, and prolonged length of ICU and hospital stay. 1-year mortality referred to the time from admission to mortality from any cause within one year. Prolonged ventilation was defined as the need for mechanical ventilation for more than 24 h. Prolonged length of stay was defined as any stay beyond the 75th percentile for the total study population. Moreover, prolonged length of ICU and hospital stays were length of stay longer than 6 days and 15 days, respectively.

### Statistical analysis

MIMIC III was regarded as the training cohort which was used to investigate whether LDH is a poor prognostic factor for patients undergoing cardiac surgery. We performed external validation in MIMIC IV to confirm the results obtained from MIMIC III. A total of 2325 patients were enrolled from MIMIC III, whereas 1387 patients were enrolled from the MIMIC IV (Additional file 1: Figure S1). Next, we compared the prognostic power of LDH with other indicators in patients undergoing cardiac surgery, including NLR, LMR, PLR, lactate and SAPS II. These indicators have previously been shown to be strong prognostic biomarkers in cardiac surgery patients. After excluding patients with missing NLR, LMR, PLR, lactate, and SAPS II data, there were 830 patients left in MIMIC III and 731 patients in MIMIC IV who were then subjected to further analysis (Additional file 1: Figure S1).

The relationship between categorical variables were examined by Pearson χ^2^ tests and reported as counts (percentage). Continuous variables were presented as means (standard deviation) or medians (range), and differences between values were examined by independent t-test or Mann–Whitney U test. Receiver operating characteristic (ROC) curve was applied to determine the best cut-off point of LDH for predicting in-hospital mortality. We performed Kaplan–Meier curves with the log-rank test to asses the one-year survival rate between groups based on the optimal cut-off value of LDH. Multivariate logistic regression analysis and Cox proportional hazards regression analysis were used to explore the predictive value of LDH for poor outcomes. Notably, LDH was tested both as a continuous and a categorical variable, and NLR, LMR, PLR, and lactate were not entered into the multivariate analysis because more than 20% of the data was missing. Significant factors associated with primary endpoint from univariate analyses were included in multivariate analysis. The following covariates in MIMIC III were adjusted as potential confounders: ethnicity, SBP, heart rate, hypertension, CHD, heart failure, BUN, SCr, sodium, potassium, glucose, SAPS II, and SOFA score (Additional file 2: Table S1). The following covariates in MIMIC IV were adjusted: hypertension, heart failure, CKD, WBC, BUN, SCr, sodium, potassium, and SAPS II (Additional file 2: Table S1). Results were expressed with odds ratios (OR) for logistic regression analysis or hazard ratio (HR) for Cox proportional hazards analysis, and their 95% confidence intervals (95% CI). To evaluate the consistency of the prognostic impact of LDH on primary endpoint, we conducted stratified analyses in different groups of gender, age, hypertension, diabetes, CHD, CKD, heart failure, and valve disease. Interaction tests between each subgroup were analyzed. We used ROC curve based on DeLong’s test to compare the predictive ability of LDH with other prognostic indicators, including NLR, LMR, PLR, lactate, and SAPS II. To assess correlations between LDH and these indicators, Pearson or Spearman analyses were performed where appropriate. All statistical analyses were examined using packages implemented in R software (version 3.6.3) and MedCalc version 19.1 (MedCalc Software, Belgium). A *p* < 0.05 was considered as statistically significant.

## Results

### Baseline characteristics of the study population

Additional file 2: Table S2 shows comparisons between baseline characteristics of patients in MIMIC III and MIMIC IV databases. Some degree of heterogeneity between the two datasets was observed. Table 1 presents the baseline characteristics of the study patients grouped by in-hospital death. In both datasets, LDH was remarkably higher in patients with in-hospital death compared to those without. Patients with in-hospital death in MIMIC III tended to be older, had faster heart rate, higher BUN, SCr, SAPS II, and SOFA score, and higher prevalence of heart failure. In addition, there were fewer white people, lower SBP, DBP, sodium, and lower prevalence of hypertension and CHD. Patients with a primary endpoint in MIMIC IV had higher proportions of heart failure and CKD, increased WBC, BUN, SCr, and SAPS II, but decreased levels of sodium and lower proportions of hypertension.

**Table 1:** Baseline clinical characteristics of patients based on in-hospital mortality

Variables	MIMIC III			MIMIC IV		
	Survivors	Non-survivors	*P*	Survivors	Non-survivors	*P*
	N = 2247	N = 78		N = 1352	N = 35	
Age, years	69.2 (60.4–77.5)	73.6 (65.0–78.9)	0.023	69.0 (61.0–77.0)	75.0 (58.5–82.0)	0.104
Male, n (%)	1516 (67.5)	50 (64.1)	0.617	945 (69.9)	22 (62.9)	0.479
Marital, n (%)			0.098			0.052
Married	1349 (60.0)	46 (59.0)		782 (57.8)	16 (45.7)	
Un-married	810 (36.0)	25 (32.1)		500 (37.0)	14 (40.0)	
Unknown	88 (3.9)	7 (9.0)		70 (5.2)	5 (14.3)	
Ethnicity, n (%)			0.043			0.193
White	1603 (71.3)	48 (61.5)		941 (69.6)	22 (62.9)	
Non-white	234 (10.4)	7 (9.0)		185 (13.7)	3 (8.6)	
Unknown	410 (18.2)	23 (29.5)		226 (16.7)	10 (28.6)	
BMI, kg/m^2^	27.6 (24.6–31.6)	28.4 (25.0–32.8)	0.218	28.8 (25.5–33.0)	30.8 (25.3–35.9)	0.152
SBP, mmHg	115 (102–128)	108 (96–126)	0.018	122 (104–140)	133 (98–153)	0.720
DBP, mmHg	59 (52–67)	56 (48–62)	0.009	65 (54–90)	66 (52–90)	0.979
Heart rate, bpm	84 (77–90)	88 (80–99)	< 0.001	80 (69–90)	80 (60–103)	0.706
Hypertension, n (%)	1330 (59.2)	30 (38.5)	< 0.001	803 (59.4)	13 (37.1)	0.014
Diabetes, n (%)	753 (33.5)	25 (32.1)	0.883	479 (35.4)	11 (31.4)	0.757
CHD, n (%)	1749 (77.8)	50 (64.1)	0.007	1059 (78.3)	25 (71.4)	0.442
Valve disease, n (%)	1109 (49.4)	45 (57.7)	0.183	680 (50.3)	22 (62.9)	0.195
Heart failure, n (%)	841 (37.4)	51 (65.4)	< 0.001	463 (34.2)	26 (74.3)	< 0.001
COPD, n (%)	27 (1.2)	2 (2.6)	0.254	12 (0.9)	0 (0.0)	1.000
CKD, n (%)	282 (12.6)	15 (19.2)	0.118	236 (17.5)	16 (45.7)	< 0.001
LDH, u/l	228 (184, 320)	436 (278–966)	< 0.001	206 (171–256)	299 (231–458)	< 0.001
WBC, k/ul	8.6 (6.8–11.6)	9.7 (7.3–13.3)	0.088	7.8 (6.4–9.7)	9.5 (7.3–13.1)	0.006
PLT, k/u	206 (161–257)	205 (148–257)	0.477	211 (172–262)	231 (176–293)	0.243
BUN, mg/dl	19 (15–26)	27 (18–42)	< 0.001	19 (15–26)	29 (19–43)	< 0.001
SCr, mg/dl	1.0 (0.8–1.3)	1.3 (1.0–1.8)	< 0.001	1.0 (0.9–1.3)	1.4 (1.1–1.9)	< 0.001
Sodium, mEq/l	139 (137–141)	138 (136–140)	0.002	139 (137–141)	138 (135–140)	0.013
Potassium, mEq/l	4.1 (3.9–4.5)	4.2 (3.9–4.9)	0.059	4.1 (3.9–4.4)	4.2 (3.9–4.8)	0.052
Glucose, mg/dl	119 (101–151)	124 (105–177)	0.092	116 (99–152)	120 (105–155)	0.234
SAPS II	36 (30–44)	46 (36–56)	< 0.001	36 (29–43)	46 (37–52)	< 0.001
SOFA score	5 (3–7)	8 (5–10)	< 0.001	2 (1–4)	3 (1–4)	0.397

*MIMIC* Medical Information Mart for Intensive Care, *BMI* body mass index, *SBP* systolic blood pressure, *DBP* diastolic blood pressure, *CHD* coronary heart disease, *COPD* chronic obstructive pulmonary disease, *CKD* chronic kidney disease, *LDH* lactate dehydrogenase, *WBC* white blood cell count, *PLT* platelets, *BUN* blood urea nitrogen, *SCr* serum creatine, *SAPS* simplified acute physiology score, *SOFA* sequential organ failure assessment

### Association between LDH and outcomes

The ROC curve presented that the optimum cut-off point of LDH for predicting in-hospital mortality in MIMIC III was > 328u/l (sensitivity 65.4%, specificity 76.6%), with an area under the curve (AUC) of 0.795 (Additional file 1: Figure S2). Next, patients were stratified into two groups (low and high LDH levels) based on the optimum LDH cut-off value. In MIMIC III, patients in the elevated LDH group showed a significantly higher risk of in-hospital and 1-year deaths, higher rates of mechanical ventilation and CRRT, and longer length of ICU and hospital stays compared to patients in the reduced LDH group (all *p* < 0.001). The results were verified in MIMIC IV (Table 2).

**Table 2:** Clinical outcomes between study cohorts

Outcomes	Low LDH (≤ 328)	High LDH (> 328)	*P*
MIMIC III	N = 1749	N = 576	
In-hospital mortality, n (%)	27 (1.5%)	51 (8.9%)	< 0.001
1-year mortality, n (%)	131 (7.5%)	107 (18.6%)	< 0.001
Prolonged ventilation, n (%)	222 (12.7)	207 (35.9%)	< 0.001
CRRT, n (%)	86 (4.9%)	77 (13.4%)	< 0.001
ICU stay > 6 days, n (%)	307 (17.6%)	264 (45.8%)	< 0.001
Hospital stay > 15 days, n (%)	356 (20.4%)	242 (42.0%)	< 0.001

MIMIC IV	N = 1196	N = 191	*P*
In-hospital mortality, n (%)	20 (1.7%)	15 (7.8%)	< 0.001
1-year mortality, n (%)	36 (3.0%)	18 (9.4%)	< 0.001
Prolonged ventilation, n (%)	181 (15.1%)	85 (44.5%)	< 0.001
CRRT, n (%)	67 (5.6%)	35 (18.3%)	< 0.001
ICU stay > 6 days, n (%)	114 (9.5%)	52 (27.2%)	< 0.001
Hospital stay > 15 days, n (%)	160 (13.4%)	83 (43.5%)	< 0.001

*MIMIC* Medical Information Mart for Intensive Care, *LDH* lactate dehydrogenase, *ICU* intensive care units, *CRRT* continuous renal replacement therapy

**Fig. 1:**
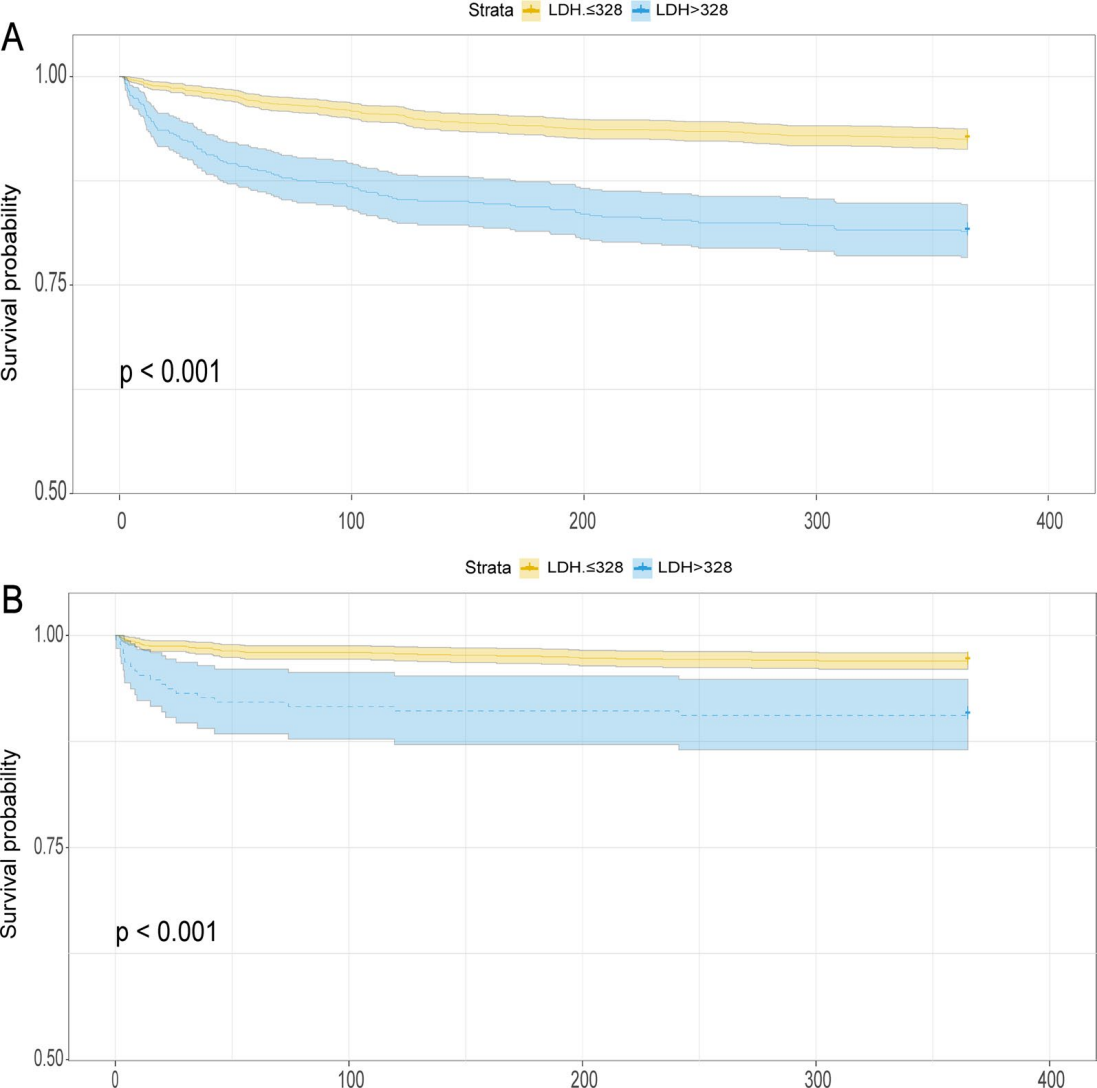
Kaplan Meier curves for 1-year mortality stratified by high and low LDH. **A** Kaplan Meier curves for 1-year mortality in MIMIC III dataset. **B** Kaplan Meier curves for 1-year mortality in MIMIC IV dataset. *LDH* lactate dehydrogenase, *MIMIC* Medical Information Mart for Intensive Care

Figure 1 presents the Kaplan–Meier curves for the 1-year cumulative survival rate according to the optimal LDH cut-off value. In MIMIC III, the incidence of 1-year death in the increased LDH (> 328) group was remarkably higher than in the decreased LDH (≤ 328) group (log-rank *p* < 0.001, Fig. 1A). Similarly, data obtained from MIMIC IV database showed that the cumulative risk of death by one year was remarkably higher in patients with high LDH (LDH > 328) than in patients with low LDH (LDH ≤ 328) (log-rank *p* < 0.001, Fig. 1B).

Multivariate regression analyses were carried out to examine the predictive potential of LDH for primary and secondary endpoints. In MIMIC III, multivariate logistic regression analyses revealed that elevated LDH was a significant independent risk factor for the occurrence of in-hospital death (*p* < 0.001, Table 3). Continuous variables of the LDH were also significantly correlated with in-hospital death in all models, despite accounting for confounding variables (*p* < 0.001, Table 3). Multivariate Cox/logistic regression analysis further demonstrated that elevated LDH were associated with an increased risk of secondary endpoints [HR (95% CI): 2.08 (1.59–2.73), *p* < 0.001 for 1-year mortality; OR (95% CI): 3.02 (2.38–3.84), *p* < 0.001 for prolonged ventilation; OR (95% CI): 2.84 (1.85–4.36), *p* < 0.001 for CRRT; OR (95% CI): 3.22 (2.58–4.02), *p* < 0.001 for ICU stay > 6 days; and OR (95% CI): 2.24 (1.80–2.80), *p* < 0.001 for hospital stay > 15 days, Table 4]. Similarly, the predictive value of LDH for adverse outcomes was verified in MIMIC IV. After accounting for confounding factors, high LDH was still a significant predictor of in-hospital mortality, whether treated LDH as a nominal or continuous variable (Table 3). Multivariate regression analysis revealed that high LDH was remarkably associated with increased risk of 1-year mortality, prolonged ventilation, CRRT, ICU stay > 6 days, and hospital stay > 15 days (all *p* < 0.05, Table 4).

**Table 3:** Predictive value of LDH for in-hospital mortality in different models

	Unadjusted		Model I		Model II	
	OR (95% Cl)	*P*	OR (95% Cl)	*P*	OR (95% Cl)	*P*
MIMIC III*						
LDH as continuous variable^a^	1.00 (1.00–1.00)	< 0.001	1.00 (1.00–1.00)	< 0.001	1.00 (1.00–1.00)	< 0.001
LDH as nominal variable^b^	6.20 (3.85–9.98)	< 0.001	4.79 (2.93–7.83)	< 0.001	4.90 (2.96–8.09)	< 0.001
MIMIC IV**						
LDH as continuous variable^a^	1.00 (1.00–1.00)	< 0.001	1.00 (1.00–1.00)	0.002	1.00 (1.00–1.00)	0.004
LDH as nominal variable^b^	5.01 (2.52–9.97)	< 0.001	3.29 (1.54–7.06)	0.002	3.80 (1.82–7.95)	< 0.001

^a^The OR was examined by per 1-point increase of LDH

^b^The OR was examined regarding the low LDH as reference

*MIMIC III

Model 1: adjusted for ethnicity, SBP, heart rate, hypertension, CHD, heart failure

Model 2: adjusted for BUN, SCr, sodium, potassium, glucose, SAPS II, SOFA score

**MIMIC IV

Model 1: adjusted for hypertension, heart_failure, CKD, WBC

Model 2: adjusted for BUN, SCr, sodium, potassium, SAPS II

*OR* odds ratio, *95% CI* 95% confidence interval, *MIMIC* Medical Information Mart for Intensive Care, *LDH* lactate dehydrogenase

**Table 4:** Predictive value of LDH for secondary endpoints

	Unadjusted		Adjusted	
	OR/HR (95% Cl)	*P*	OR/HR(95% Cl)	*P*
MIMIC III^a^				
1-year mortality	2.69 (2.08–3.47)	< 0.001	2.08 (1.59–2.73)	< 0.001
Prolonged ventilation	3.86 (3.09–4.81)	< 0.001	3.02 (2.38–3.84)	< 0.001
CRRT	2.98 (2.16–4.12)	< 0.001	2.84 (1.85–4.36)	< 0.001
ICU stay > 6 days	3.97 (3.24–4.88)	< 0.001	3.22 (2.58–4.02)	< 0.001
Hospital stay > 15 days	2.84 (2.32–3.47)	< 0.001	2.24 (1.80–2.80)	< 0.001
MIMIC IV^b^				
1-year mortality	3.27 (1.86–5.76)	< 0.001	1.99 (1.05–3.76)	0.034
Prolonged ventilation	4.50 (3.25–6.23)	< 0.001	2.77 (1.90–4.04)	< 0.001
CRRT	3.78 (2.43–5.88)	< 0.001	2.92 (1.57–5.40)	0.001
ICU stay > 6 days	3.55 (2.45–5.15)	< 0.001	2.37 (1.55–3.63)	< 0.001
Hospital stay > 15 days	4.98 (3.57–6.93)	< 0.001	3.05 (2.09–4.46)	< 0.001

The OR/HR was examined regarding the low LDH as reference

*OR* odds ratio, *HR* hazard ratio, *95% CI* 95% confidence interval, *MIMIC* Medical Information Mart for Intensive Care, *LDH* lactate dehydrogenase, *ICU* intensive care units, *CRRT* continuous renal replacement therapy

^a^The baseline model includes variables that are significant in univariate logistic proportional hazard analysis in MIMIC III, including ethnicity, SBP, heart rate, hypertension, CHD, heart failure, BUN, SCr, sodium, potassium, glucose, SAPS II, SOFA score(details shown in Additional file 2: Table S1)

^b^The baseline model includes variables that are significant in univariate logistic proportional hazard analysis in MIMIC IV, including hypertension, heart failure, CKD, WBC, BUN, SCr, sodium, potassium, SAPS II (details shown in Additional file 2: Table S1)

### Subgroup analyses

**Fig. 2:**
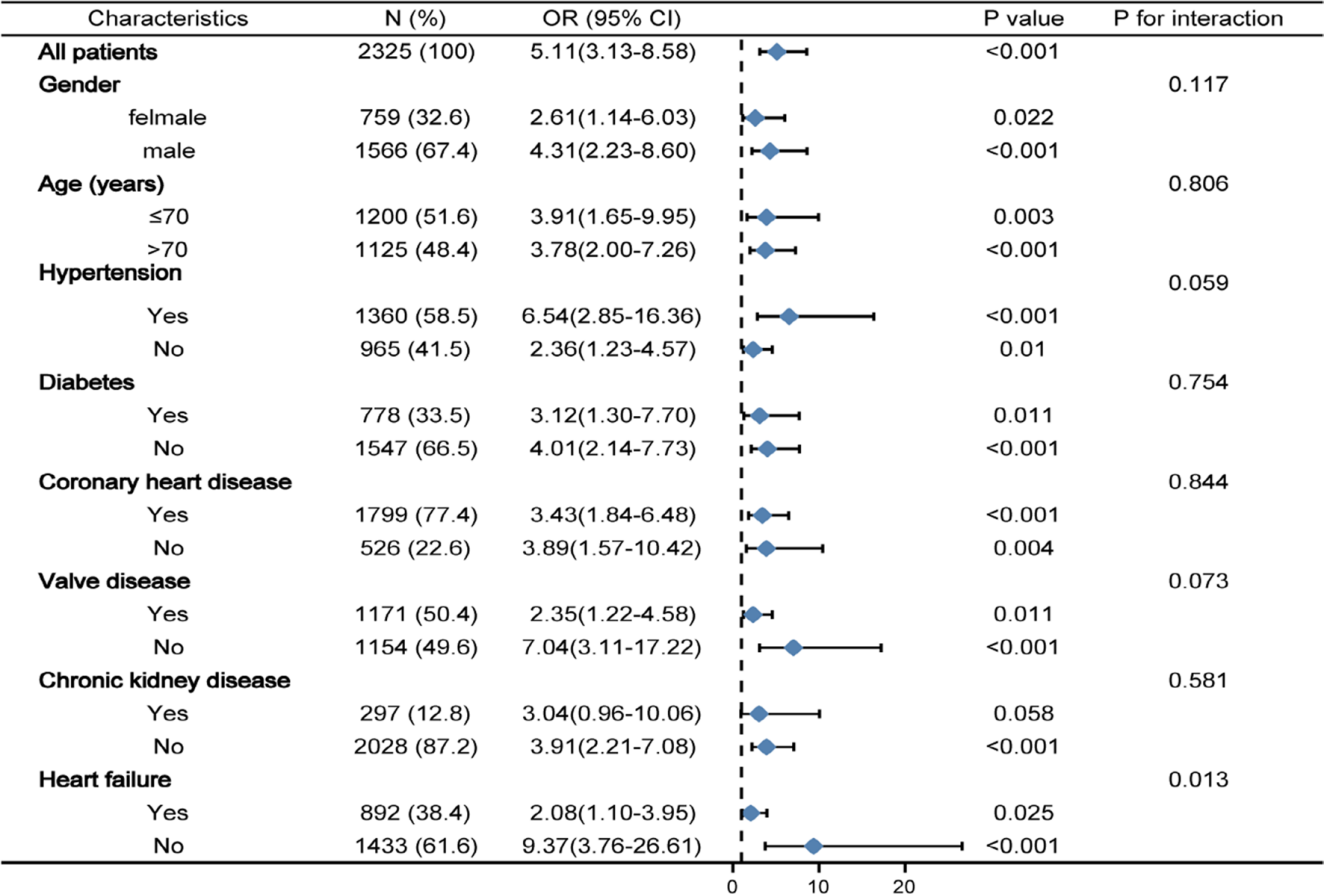
Logistic regression analysis evaluating prognostic value of LDH in various subgroups in MIMIC III. The OR was examined regarding the low LDH as reference. *LDH* lactate dehydrogenase, *OR* odds ratio, *95% CI* 95% confidence interval, *MIMIC* Medical Information Mart for Intensive Care

Further analyses was performed to identify risk stratification of LDH for primary endpoint in various subgroups (Fig. 2). Elevated LDH was consistently correlated with primary endpoint in various subgroups, including female or male, age ≤ 70 or > 70 years, with or without hypertension, diabetes, CHD, CKD, heart failure, and valve disease. It is worth noting that the predictive implication of LDH seemed to be more prominent in patients without heart failure (*P*_interaction_ = 0.013).

### Comparison with other indicators

**Fig. 3:**
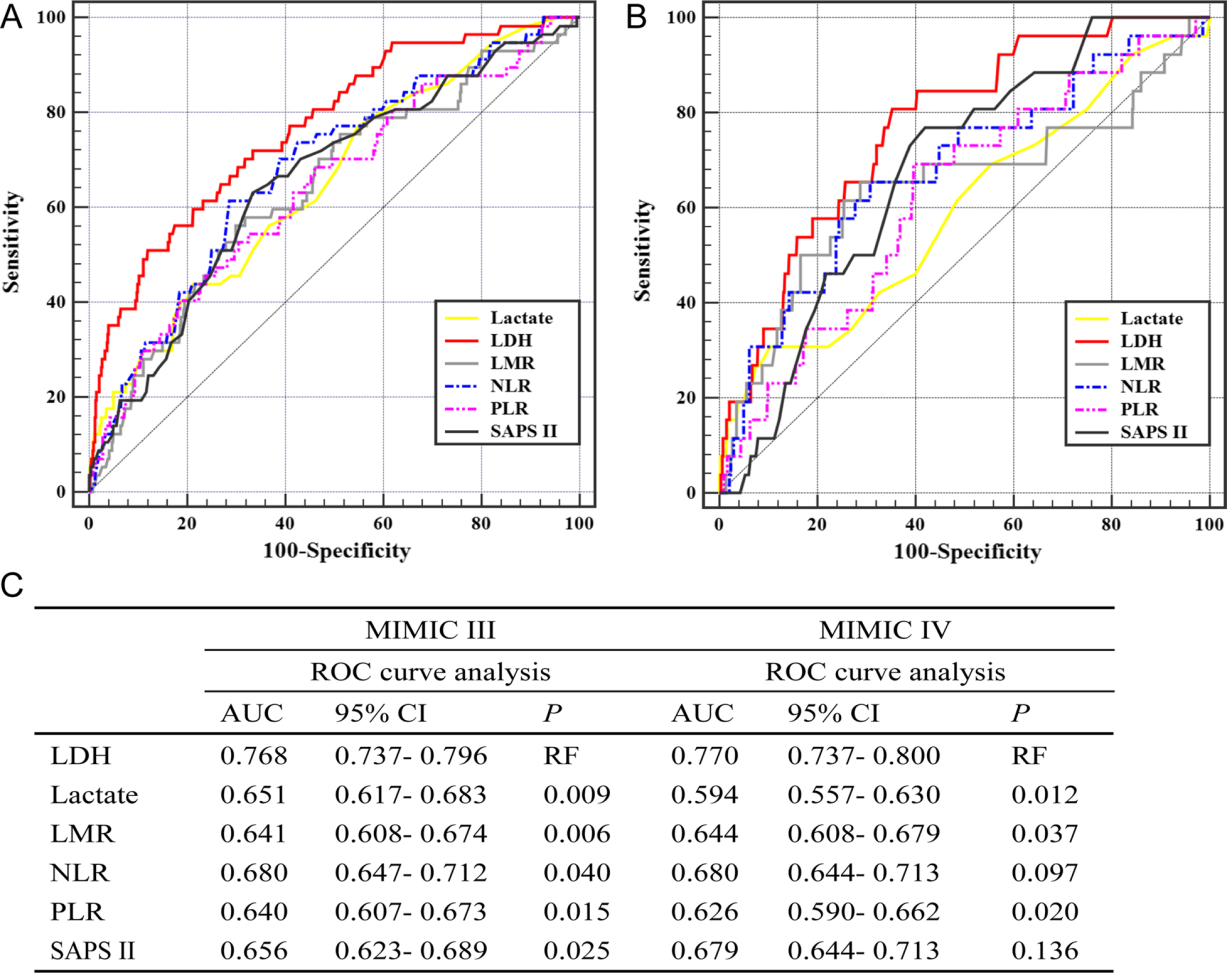
Predictive performance of various indicators for in-hospital mortality. **A** ROC curve of LDH, Lactate, LMR, NLR, PLR and SAPS II in MIMIC III dataset for predicting in-hospital mortality. **B** ROC curve of LDH, Lactate, LMR, NLR, PLR and SAPS II in MIMIC IV dataset for predicting in-hospital mortality. **C** The predictive power of LDH was compared with the other objective indices using ROC curve analysis. *ROC* receiver operator characteristic, *AUC* area under the curve, *95% CI* 95% confidence interval, *LDH* lactate dehydrogenase, *NLR* neutrophil-lymphocyte ratio, *LMR* lymphocyte-monocyte ratio, *PLR* platelet-lymphocyte ratio, *SAPS* simplified acute physiology score, *MIMIC* Medical Information Mart for Intensive Care

The C-statistic was performed to compare the predictive power of various indicators for in-hospital mortality. Results showed that LDH yielded the highest AUC values compared to other indicators in both MIMIC III and IV (Fig. 3A, B). The Delong test found a significant difference between the AUCs of LDH and other indicators, including NLR, LMR, PLR, lactate, and SAPS II in MIMIC III (all *p* < 0.05, Fig. 3C). Comparison of the AUCs between LDH and PLR, LMR, and lactate showed significant differences in MIMIC IV (*p* < 0.05, Fig. 3C). Moreover, Spearman or Pearson tests were implemented to assess the correlation between LDH and the above indicators (Additional file 2: Table S3). Results indicated that LDH was positively related to lactate, NLR, and SAPS II, but negatively related to LMR.

## Discussion

We are the first to investigate the prognostic value of LDH for poor prognosis in cardiac surgery patients. The following are the major findings of the study: (1) patients in the high LDH group had significantly higher incidence of primary and secondary endpoints compared to patients in the low LDH group; (2) elevated LDH at admission was independently correlated with increased risk of primary and secondary endpoints, and this finding persisted even after adjusting for confounding risk factors; and (3) LDH showed better predictive value for primary endpoint than other indicators, including NLR, LMR, PLR, lactate, and SAPS II. Notably, the above findings were fully demonstrated in the MIMIC IV database, implying the reliability of our results.

Previous studies have detected that elevated LDH level is correlated with unfavorable outcomes in patients with acute decompensated heart failure, acute aortic syndromes, and acute aortic dissection [18–20]. Moreover, higher LDH was found to be related to increased risk of cardiovascular mortality in patients with chronic arsenic exposure [21] or incident dialysis [22]. However, little is known regarding LDH and cardiac surgery. Our study demonstrated that elevated LDH at admission was a vital marker for predicting mortality in cardiac surgery patients. Previous investigations demonstrated that complications after cardiac surgery are related to increased risk of operative death and prolonged hospital stay [5, 23]. Thus, to better elucidate the correlation between LDH and unfavorable outcomes in cardiac surgery patients, we set CRRT, prolonged ventilation, and prolonged length of ICU and hospital stay as the secondary endpoints. Multivariate analysis results also revealed that LDH was an independent predictor of secondary endpoints.

Next, we compared the prognostic value of NLR, LMR, PLR, lactate, SAPS II, and LDH for predicting in-hospital death in cardiac surgery patients. NLR and LMR have previously been shown to be strong prognostic biomarkers in cardiac surgery patients [10, 11, 24]. To date, there is no direct evidence to prove that PLR is correlated with cardiac surgery prognosis. However, a previous study found that PLR is correlated with increased surgical risk in patients undergoing transcatheter aortic-valve replacement [25]. Studies have also revealed that there is an important relationship between PLR and clinical prognosis in patients with CHD [26] or acute coronary syndrome [27]. These findings may potentially suggest that there are certain associations between PLR and cardiac surgery prognosis. Prior evidences have demonstrated that elevated lactate levels are prominently correlated with increased risk of unfavorable outcomes after cardiac surgery [28, 29]. Schoe et al. [30] found that SAPS II had better discriminatory power than SOFA score for predicting mortality in cardiac surgery patients. Herein, LDH showed a better performance for predicting in-hospital mortality in cardiac surgery patients compared to the above prognostic indicators.

The underlying mechanisms that may account for the relationship between LDH and adverse cardiac surgery outcomes may be attributed to the following reasons: 1) LDH is involved in anaerobic glycolysis and 2) LDH is a marker of inflammation. On the one hand, CHD is closely related to insufficient supply of blood to the myocardium, which leads to cardiomyocytes ischemia and hypoxia. Moreover, there may be a mutual cause–effect association between hypoxia and heart valve disease: mid to late valve disease is accompanied by intracardiac hypoxia; in turn, hypoxia further aggravates the progression of disease [31]. In ischemic tissues, LDH dependent glycolysis is the main source of ATP in cells [32]. Previous studies revealed that hypoxia and apoptosis of cardiomyocytes can induce LDH expression [12, 33]. Our analysis also found that LDH had a positive relationship with SAPS II, which suggests that the LDH level represents disease severity. Inflammation might be additional possible explanation for the association between increased LDH level and adverse prognosis in patients after cardiac surgery. Accumulating evidence revealed that inflammatory biomarkers, such as NLR [34, 35] and CRP [36], have been reported to be correlated with adverse outcomes of CHD or acute myocardial infarction. There is a paucity of reports about the prognostic effect of inflammatory markers in valve disease. Previous studies have provided evidences of LDH as an inflammation indicator in lung diseases [37], acute pancreatitis [38], and cancer[39]. Song et al. [40] found that inhibiting the expression of LDH had an anti-inflammatory effect through the downregulation of inflammatory mediators. Collectively, these findings suggest that serum LDH is closely related to inflammation. Besides, this study found that LDH showed a positive association with inflammatory markers, including LMR and NLR.

However, this study was subject to some limitations that must be acknowledged. First, since the half-life of LDH is long, it not reflects the dynamic changes of the disease. Second, we did not include NLR, LMR, PLR, and lactate into multivariate regression analysis due to missing data. Third, MIMIC database lacks some important information, including intra- and post-operative information, EuroSCORE II and types of surgery, which have been demonstrated to influence the rate of operative mortality after cardiac surgery.

## Conclusion

Elevated preoperative LDH may be a robust predictor of poor prognosis in cardiac surgery patients, and provides a more powerful value to predict in-hospital mortality than NLR, LMR, PLR, lactate, and SAPS II.

## Supplementary Information

**Additional file 1.** **Supplementary Figure 1.** Flow chart of the study population enrollment.

**Additional file 2.** **Supplementary table 1.** Univariate regression analyses for in-hospital mortality in MIMIC III and MIMIC IV. **Supplementary table 2.** Characteristics of the study population. **Supplementary table 3.** Correlation between LDH and other prognostic indicators.

## Data Availability

All data in this study was extracted from MIMIC III (https://mimic.mit.edu/docs/iii/) and MIMIC IV (https://mimic.mit.edu/docs/iv/) databases. The datasets used or analyzed during the current study are available from the corresponding author on reasonable request.

## Abbreviations

AUCs: Area under curves
BMI: Body mass index
BUN: Blood urea nitrogen
CABG: Coronary artery bypass graft
CHD: Coronary heart disease
CI: Confidence interval
CKD: Chronic kidney disease
COPD: Chronic obstructive pulmonary disease
CPB: Cardiopulmonary bypass
CRRT: Continuous renal replacement therapy
DBP: Diastolic blood pressure
HF: Heart failure
HR: Hazard ratio
ICU: Intensive care unit
LMR: Lymphocyte-monocyte ratio
MIMIC IV: Medical Information Mart for Intensive Care IV
NLR: Neutrophil–lymphocyte ratio
OR: Odds ratio
PLR: Platelet–lymphocyte ratio
PLT: Platelet
ROC: The receiver operating characteristic curves
SAPS: Simplified acute physiology score
SBP: Systolic blood pressure
SCr: Serum creatine
SOFA: The sequential organ failure assessment
WBC: White blood cell
