# Supplementary material for: Impact of lactate dehydrogenase on prognosis of patients undergoing cardiac surgery
Source: BMC Cardiovasc Disord. 2022 Sep 10;22:404. doi: 10.1186/s12872-022-02848-7 (PMC9463775; doi:10.1186/s12872-022-02848-7)
Supplement: Supplementary file 1 — Additional file 1. Supplementary Figure 1. Flow chart of the study population enrollment. [file 12872_2022_2848_MOESM1_ESM.docx]

**
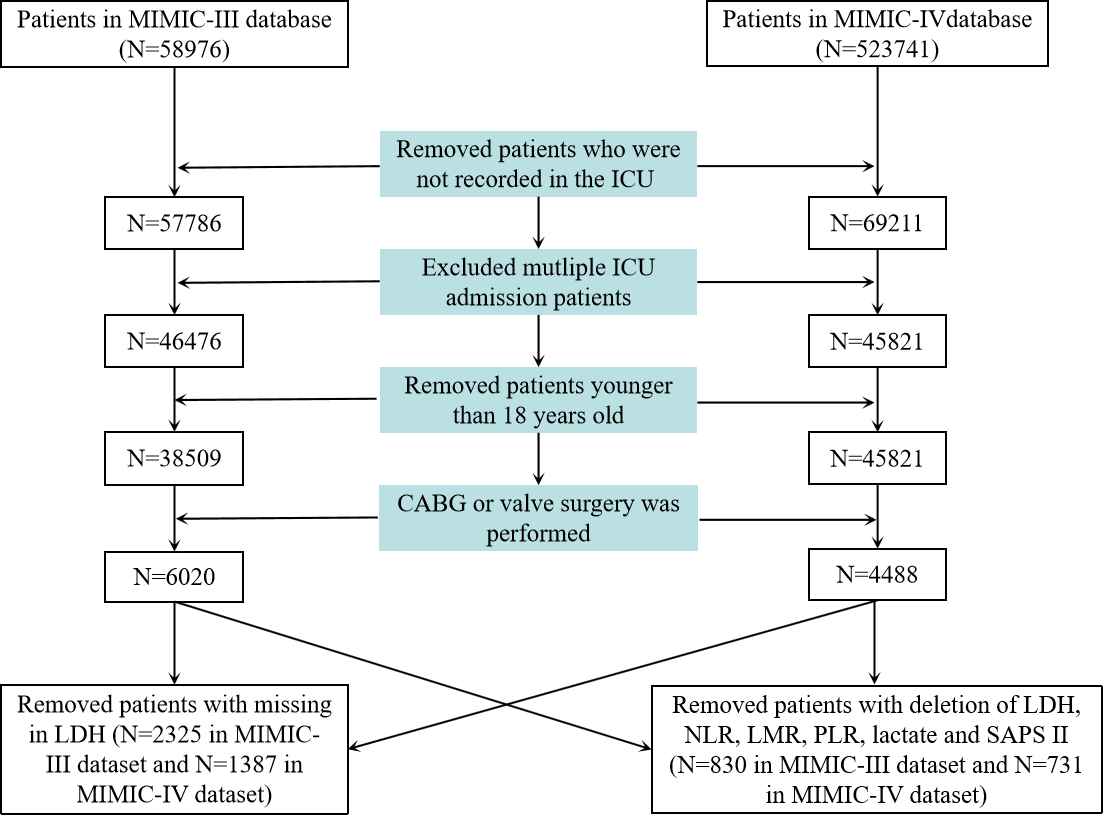
**

**Supplementary Figure 1.** Flow chart of the study population enrollment**.**

MIMIC, Medical Information Mart for Intensive Care; ICU, intensive care unit; CABG, coronary artery bypass graft surgery; LDH, lactate dehydrogenase; NLR, neutrophil-lymphocyte ratio; LMR, lymphocyte-monocyte ratio; PLR, platelet-lymphocyte ratio; SAPS, simplified acute physiology score.


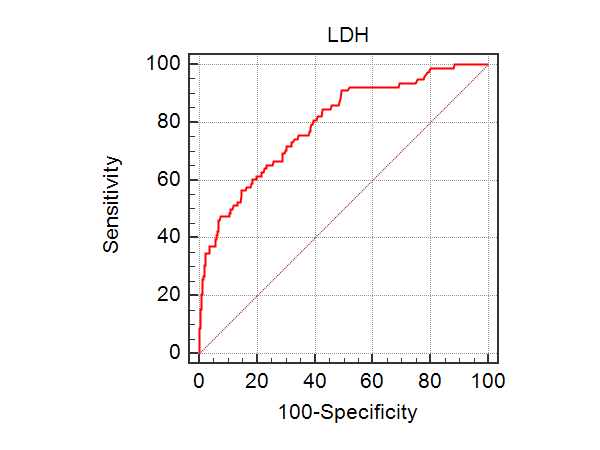


**Supplementary Figure 2.** The receiver operating characteristic curve of LDH for predicting in-hospital mortality in MIMIC III. The best cut-off value was > 328u/l. Area under the curve was 0.795 and the sensitivity, specificity, 95%CI lower and 95%CI upper were 65.4%, 76.6%, 0.778 and 0.811, respectively. LDH, lactate dehydrogenase; 95% CI, 95% confidence interval; MIMIC, Medical Information Mart for Intensive Care.

.
