# Supplementary material for: Impact of lactate dehydrogenase on prognosis of patients undergoing cardiac surgery
Source: BMC Cardiovasc Disord. 2022 Sep 10;22:404. doi: 10.1186/s12872-022-02848-7 (PMC9463775; doi:10.1186/s12872-022-02848-7)
Supplement: Supplementary file 2 — Additional file 2. Supplementary table 1. Univariate regression analyses for in-hospital mortality in MIMIC III and MIMIC IV. Supplementary table 2. Characteristics of the study population. Supplementary table 3. Correlation between LDH and other prognostic indicators. [file 12872_2022_2848_MOESM2_ESM.doc]

**Supplementary table 1** Univariate regression analyses for in-hospital mortality in MIMIC III and MIMIC IV.

|  | MIMIC III | | MIMIC IV | |
| --- | --- | --- | --- | --- |
|  | OR (95%CI) | *P* | OR (95%CI) | *P* |
| Age, years | 1.02 (0.99-1.04) | 0.054 | 1.01 (0.98-1.04) | 0.341 |
| Male, n (%) | 0.86 (0.54-1.38) | 0.533 | 0.73 (0.36-1.46) | 0.373 |
| Marital, n (%) |  | 0.092 |  | 0.060 |
| Married | RF |  | RF |  |
| Un-married | 0.91 (0.55-1.48) | 0.693 | 1.37 (0.66-2.83) | 0.397 |
| Unknown | 2.33 (1.02-5.32) | 0.044 | 3.49 (1.24-9.81) | 0.018 |
| Ethnicity, n (%) |  | 0.047 |  | 0.170 |
| White | RF |  |  |  |
| Non-white | 0.99 (0.45-2.23) | 0.998 | 0.69 (0.21-2.34) | 0.556 |
| Unknown | 1.87 (1.13-3.12) | 0.016 | 1.89 (0.88-4.05) | 0.101 |
| BMI, kg/m^2^ | 1.03 (0.99-1.07) | 0.071 | 1.04 (0.99-1.09) | 0.086 |
| SBP, mmHg | 0.98 (0.97-0.99) | 0.008 | 1.00 (0.99-1.02) | 0.992 |
| DBP, mmHg | 0.97 (0.95-0.99) | 0.002 | 0.99 (0.98-1.02) | 0.964 |
| Heart rate, bpm | 1.04 (1.02-1.05) | <0.001 | 1.01 (0.99-1.02) | 0.412 |
| Hypertension, n (%) | 0.43 (0.27-0.68) | <0.001 | 0.40 (0.20-0.81) | 0.010 |
| Diabetes, n (%) | 0.94 (0.58- 1.52) | 0.788 | 0.84 (0.41-1.72) | 0.625 |
| CHD, n (%) | 0.51 (0.32-0.82) | 0.005 | 0.69 (0.33-1.46) | 0.332 |
| Valve disease, n (%) | 1.39 (0.89-2.21) | 0.149 | 1.67 (0.84-3.35) | 0.146 |
| Heart failure, n (%) | 3.16 (1.97-5.07) | <0.001 | 5.55 (2.58-11.93) | <0.001 |
| COPD, n (%) | 2.16 (0.51-9.27) | 0.298 | 0.00 (0.00-0.00) | 0.999 |
| CKD, n (%) | 1.66 (0.93-2.95) | 0.085 | 3.98 (2.02-7.86) | <0.001 |
| LDH, u/l | 1.00 (1.00-1.00) | <0.001 | 1.00 (1.00-1.00) | <0.001 |
| WBC, k/ul | 1.04 (0.99-1.08) | 0.100 | 1.09 (1.03-1.14) | 0.003 |
| PLT, k/u | 0.99 (0.99-1.00) | 0.802 | 1.00 (0.99-1.01) | 0.191 |
| BUN, mg/dl | 1.03 (1.02-1.04) | <0.001 | 1.03 (1.02-1.05) | <0.001 |
| SCr, mg/dl | 1.25 (1.12-1.39) | <0.001 | 1.33 (1.13-1.56) | 0.001 |
| Sodium, mEq/l | 0.89 (0.84-0.96) | 0.001 | 0.88 (0.79-0.96) | 0.006 |
| Potassium, mEq/l | 1.52 (1.09-2.12) | 0.015 | 2.02 (1.13-3.59) | 0.017 |
| Glucose, mg/dl | 1.00 (1.00-1.01) | 0.002 | 1.00 (0.99-1.01) | 0.066 |
| SAPS II | 1.05 (1.03-1.07) | <0.001 | 1.04 (1.02-1.07) | <0.001 |
| SOFA score | 1.27 (1.18-1.36) | <0.001 | 1.08 (0.95-1.22) | 0.262 |

OR, odds ratio; 95%CI, 95% confidence interval; MIMIC, Medical Information Mart for Intensive Care; BMI, body mass index; SBP, systolic blood pressure; DBP, diastolic blood pressure; CHD, coronary heart disease; COPD, chronic obstructive pulmonary disease; CKD, chronic kidney disease; LDH, lactate dehydrogenase; WBC, white blood cell count; PLT, platelets; BUN, blood urea nitrogen; SCr, serum creatine; SAPS, simplified acute physiology score; SOFA, sequential organ failure assessment.

**Supplementary table 2** Characteristics of the study population.

| Variables | MIMIC-III | MIMIC-IV | *P* |
| --- | --- | --- | --- |
|  | N=2325 | N=1387 |  |
| Age, years | 69.4 (60.5, 77.5) | 69.0 (61.0, 78.0) | 0.761 |
| Male, n (%) | 1566 (67.4%) | 967 (69.7%) | 0.144 |
| Marital, n (%) |  |  | 0.104 |
| Married | 1395 (60.0%) | 798 (57.5%) |  |
| Un-married | 835 (35.9%) | 514 (37.1%) |  |
| Unknown | 95 (4.1%) | 75 (5.4%) |  |
| Ethnicity, n (%) |  |  | 0.010 |
| White | 1651 (71.0%) | 963 (69.4%) |  |
| Non-white | 241 (10.4%) | 188 (13.6%) |  |
| Unknown | 433 (18.6%) | 236 (17.0%) |  |
| BMI, kg/m^2^ | 27.7 (24.6, 31.6) | 28.8 (25.5, 33.0) | < 0.001 |
| SBP, mmHg | 114 (102, 128) | 122 (104, 140) | < 0.001 |
| DBP, mmHg | 58 (51, 67) | 65 (54, 90) | < 0.001 |
| Heart rate, bpm | 84 (77, 90) | 80 (69, 91) | < 0.001 |
| Hypertension, n (%) | 1360 (58.5%) | 816 (58.8%) | 0.867 |
| Diabetes, n (%) | 778 (33.5%) | 490 (35.3%) | 0.261 |
| CHD, n (%) | 1799 (77.4%) | 1084 (78.2%) | 0.610 |
| Valve disease, n (%) | 1154 (49.6%) | 702 (50.6%) | 0.587 |
| Heart failure, n (%) | 892 (38.4%) | 489 (35.3%) | 0.063 |
| COPD, n (%) | 29 (1.2%) | 12 (0.9%) | 0.360 |
| CKD, n (%) | 297 (12.8%) | 252 (18.2%) | < 0.001 |
| LDH, u/l | 231 (186, 328) | 207 (172, 259) | < 0.001 |
| WBC, k/ul | 8.6 (6.8, 11.6) | 7.8 (6.4, 9.7) | < 0.001 |
| PLT, k/u | 206.0 (161.0, 257.0) | 211.5 (172.0, 262.8) | 0.004 |
| BUN, mg/dl | 19 (15, 26) | 19 (15, 26) | 0.070 |
| SCr, mg/dl | 1.0 (0.8, 1.3) | 1.0 (0.9, 1.3) | 0.474 |
| Sodium, mEq/l | 139 (137, 141) | 139 (137, 141) | 0.339 |
| Potassium, mEq/l | 4.1 (3.9, 4.5) | 4.1 (3.9, 4.4) | 0.007 |
| Glucose, mg/dl | 119 (101, 152) | 116 (99, 152) | 0.078 |
| SAPS II | 36 (30, 45) | 36 (29, 44) | 0.030 |
| SOFA score | 5 (3, 7) | 2 (1, 4) | < 0.001 |
| CABG, n (%) | 1723 (74.1%) | 1015 (73.2%) | 0.560 |
| Valve surgery, n (%) | 1016 (43.7%) | 603 (43.5%) | 0.921 |
| CABG + valve surgery, n (%) | 414 (17.8%) | 231 (16.7%) | 0.395 |
| In-hospital mortality, n (%) | 78 (3.4%) | 35 (2.5%) | 0.184 |
| 1-year mortality, n (%) | 238 (10.2%) | 54 (3.9%) | < 0.001 |
| Prolonged ventilation, n (%) | 429 (18.5%) | 266 (19.2%) | 0.613 |
| CRRT, n (%) | 163 (7.0%) | 102 (7.4%) | 0.744 |
| Length of ICU stay, days | 3.1 (1.8, 6.0) | 2.2 (1.3, 3.5) | < 0.001 |
| Length of hospital stay, days | 10.0 (7.1, 15.3) | 8.9 (6.9, 12.9) | < 0.001 |

MIMIC, Medical Information Mart for Intensive Care; BMI, body mass index; SBP, systolic blood pressure; DBP, diastolic blood pressure; CHD, coronary heart disease; COPD, chronic obstructive pulmonary disease; CKD, chronic kidney disease; LDH, lactate dehydrogenase; WBC, white blood cell count; PLT, platelets; BUN, blood urea nitrogen; SCr, serum creatine; SAPS, simplified acute physiology score; SOFA, sequential organ failure assessment; CABG, coronary artery bypass graft surgery; CRRT, continuous renal replacement therapy; ICU, intensive care units.

**Supplementary table 3** Correlation between LDH and other prognostic indicators.

|  | LDH | |
| --- | --- | --- |
|  | Correlation coefficient | *P* |
| MIMIC III |  |  |
| Lactate | 0.270 | <0.001 |
| LMR | -0.220 | <0.001 |
| NLR | 0.300 | <0.001 |
| PLR | -0.031 | 0.379 |
| SAPS II | 0.110 | 0.002 |
| MIMIC IV |  |  |
| Lactate | 0.110 | 0.003 |
| LMR | -0.290 | <0.001 |
| NLR | 0.280 | <0.001 |
| PLR | -0.007 | 0.851 |
| SAPS II | 0.150 | <0.001 |

LDH, lactate dehydrogenase; LMR, lymphocyte-monocyte ratio; NLR, neutrophil-lymphocyte ratio; PLR, platelet-lymphocyte ratio; SAPS, simplified acute physiology; MIMIC, Medical Information Mart for Intensive Care.
